# Energy availability influences the dynamics of thermal phenotypic plasticity

**DOI:** 10.1242/jeb.251713

**Published:** 2026-01-14

**Authors:** Martine Camilla Graham, Tim Burton, Sigurd Einum

**Affiliations:** ^1^ Department of Biology, Norwegian University of Science and Technology, Realfagbygget, 7491 Trondheim, Norway; ^2^ Norwegian Institute for Nature Research, Høgskoleringen 9, 7034 Trondheim, Norway

**Keywords:** Acclimation capacity, Acclimation rate, CT_max_, Critical temperature, Speed of plasticity, Rate of plasticity, Reaction norm, Thermal tolerance, Timescale of plasticity, Food abundance

## Abstract

We tested whether food availability limits phenotypic plasticity in thermal tolerance in the amphipod *Echinogammarus marinus*. We shifted specimens from 10°C to an acclimation temperature of 20°C, and kept them there for different durations with and without food before measuring the time to immobilization at 30°C. Our results show that thermal tolerance increases with acclimation duration, but this response was about two times more pronounced in fed than in unfed individuals. We also decomposed the plastic response into a rate component (how fast the trait changes) and a capacity component (by how much it changes). This showed that the overall effect of food treatment on the temporal dynamics of thermal tolerance was primarily driven by the effect on capacity. We conclude that laboratory derived thermal tolerance data from experiments where ecological conditions are otherwise optimal may provide overly optimistic estimates of how well organisms deal with extreme events through phenotypic plasticity.

## Teaser

**Summary:** Energetic status influences plasticity capacity in thermal tolerance, but not plasticity rate. Therefore, the ability of wild organisms to deal with extreme events through plasticity may be less pronounced than that inferred from laboratory experiments.

## INTRODUCTION

Adaptive phenotypic plasticity is a phenomenon common to all organisms that enables the expressed phenotype of an individual to track changes in the local optimum phenotype. Consequently, phenotypic plasticity allows organisms to shift their phenotypes in response to environmental change in a way that aims to minimize the fitness loss or maximize the fitness gain. Phenotypic plasticity is therefore crucial if organisms are to maintain fitness when environments fluctuate (Lande, 2014; Botero et al., 2015; Tufto, 2015). Of the plethora of environmental variables that tend to fluctuate across temporal scales, temperature stands out in importance. The majority of animal taxa (i.e. ectotherms) have a limited ability to regulate their body temperature independently of their surroundings. Hence, fluctuations in temperature may increasingly challenge the fitness of this group of organisms as high-temperature events are predicted to become both more frequent and of a greater magnitude in the near future (van der Wiel and Bintanja, 2021). Fortunately, many ectothermic organisms show plastic responses that may reduce their vulnerability to fluctuations in temperature. For example, laboratory experiments show that prior acclimation to sublethal warm temperatures increases tolerance of acute heat exposure (Burton and Einum, 2025). A similar pattern has been observed in nature, where tolerance of acute temperature exposure has been observed to change over both long (seasonal) and short (days) time scales in response to changes in environmental temperature (Noer et al., 2022; Sasaki et al., 2025).

The plastic adjustment of thermal tolerance in response to sub-lethal temperature exposure may have evolved for two reasons. First, in an autocorrelated stochastic environment, experiencing high but sub-lethal temperatures might provide a reliable clue about the probability of experiencing lethal temperature in the near future. Second, when acclimating to a new but sub-lethal temperature, organisms adjust their underlying physiology so that their overall performance is maximized. A side effect of this general acclimation response is that the performance curve is also elevated around the new temperature (Baldwin and Hochachka, 1970; Berry and Bjorkman, 1980), which may by itself result in an increase in thermal tolerance. Either way, producing the requisite changes in underlying physiology presumably come at an energetic cost, which sets an upper limit to the evolved response (DeWitt et al., 1998). The cost associated with producing a plastic or acclimation response raises the question of whether it may be compromised under energy limitation. If so, the fitness effects of temperature fluctuations can be expected to depend on ecological conditions that can affect the energy intake of individuals, such as population density, parasites, disease and food availability. Few studies have addressed this question, and those that have show conflicting results. For example, in the Parthenium beetle (*Zygogramma bicolorata*), positive effects of acclimation to a warm temperature on thermal tolerance were only observed in individuals that had been fed prior to the experiment, suggesting that energy limitation prevented a plastic response in starved individuals (Chidawanyika et al., 2017). In contrast, in the zooplankton *Daphnia magna*, no effect of feeding treatment on the plasticity of thermal tolerance was observed, and in fact, the nutrition deprived treatment had a higher thermal tolerance (independent of acclimation treatment) than the non-deprived treatment (Yampolsky et al., 2014). Thus, more studies are required to establish the effect of energy availability on the expression of phenotypic plasticity.

Another aspect concerning the energy limitation of phenotypic plasticity that has received scant attention is the relative importance of this for the capacity versus the rate of plasticity (for illustration of these aspects of plasticity, see fig. 1 in Einum and Burton, 2023). Changes in a given environmental variable can be described by two separate axes: amplitude and time. Hence, the ability of an individual to produce the phenotypic change required to match the current local optimum will depend both on how much and how rapidly a change in the phenotype can occur. How much the phenotype can change is represented by the capacity for plasticity, which describes the magnitude of change in phenotype per unit change in the environment (i.e. the reaction norm slope). This first dimension of phenotypic plasticity has been well studied both theoretically and empirically (Padilla and Adolph, 1996; Gabriel, 2005; Gabriel et al., 2005; Lande, 2009, 2014; Seebacher et al., 2015; Siljestam and Östman, 2017; Einum et al., 2019; Morgan et al., 2022; Pottier et al., 2022). The capacity for plasticity determines to what extent matching between the realized and optimum phenotype occurs for a given amplitude of environmental change once full phenotypic adjustment has been achieved. In comparison, far less conceptual and empirical focus has fallen upon the question of how rapidly such phenotypic adjustment can occur. However, the rate of phenotypic plasticity will also influence to what extent the expressed phenotype matches the optimum. This is because this rate determines the extent to which the change in the expressed phenotype lags behind the change in the local optimum (Fey et al., 2021). Thus, if energy limitation impacts the expression of phenotypic plasticity, it may affect fitness trough both the capacity for and the rate of plasticity. However, studies that account for the temporal dynamics of environments and traits and decompose energy limitation effects into these two components are still lacking.

To examine how energy limitation affects the dynamics of phenotypic plasticity, we performed laboratory experiments using the amphipod species *Echinogammarus marinus* (Gammaridae). Gammaridae are keystone species because they are abundant, perform a critical role in decomposing organic matter, and are a major source of food for fish and invertebrate predators (Hieber and Gessner, 2002; Semsar-Kazerouni and Verberk, 2018). They are also known to show a decrease in thermal tolerance in response to starvation (Semsar-Kazerouni et al., 2020), but it remains unknown how the acclimation response depends on food availability. We acclimated individuals to high temperature for varying periods of time in the presence and absence of food before measuring their ability to tolerate exposure to a higher temperature. Then, using a recently developed analytical approach (Burton and Einum, 2025), we were able to test the hypothesis that energy limitation acts on the rate and/or capacity components of phenotypic plasticity.

## MATERIALS AND METHODS

### Animal collection and cultivation

Individuals of *Echinogammarus marinus* (Leach 1816) were collected in Korsvika in Trondheimsfjorden, Norway (63°26′58.7″N, 10°25′54.2″E), in January 2024. The amphipods were collected from underneath rocks in the intertidal zone at low tide. Upon collection, the specimens were transferred to the lab and kept in a 50 liter cultivation tank for 2 weeks prior to the experiment. They were maintained at a constant temperature of 10°C and a salinity of 15‰, under a 24 h:0 h light:dark regime. Based on typical acclimation rates of thermal tolerance in aquatic ectotherms (Einum and Burton, 2023), as well as those observed in the present study (see Results and Discussion), a 2-week acclimation should ensure that full acclimation is approached. The cultivation tank was equipped with an aquarium air pump providing aeration, and rocks from the collection site to provide shelter. Fish feed (tetra cichlid colour) was provided three times per week, and the tank water was replaced completely once per week.

### Acclimation and thermal tolerance assays

Acclimation to a sublethal high temperature (20°C) was conducted in jars containing 0.2 liters of 15‰ saltwater in constant dim light, with daily water replacement. To produce organisms that had experienced different lengths of acclimation to 20°C prior to the measurement of thermal tolerance, groups of individuals were abruptly transferred from 10°C to 20°C at different times (96, 48, 24, 12 and 0 h before exposure) prior to thermal tolerance assays at 30°C. No mortality was observed during acclimation. The choice of 30°C for the thermal tolerance assay was based on pilot experiments demonstrating that this temperature would result in cessation of movement over a suitable time period (minutes to a few hours). The experiment was run in two parallel treatments, where the fed treatment received fish feed (tetra cichlid colour) for 4 h every day for 96 h prior to exposure to 30°C, and the unfed treatment received no food. Thermal tolerance was then quantified in 6 runs (replicates over time), with up to 5 individuals from each of the 10 treatments (five acclimation durations×two food treatments) in each run (i.e. maximum 30 individuals from each treatment, total of 253 individuals).

The thermal tolerance assay was conducted following the method of Burton et al. (2020). Specimens of *E. marinus* were put into individual wells in a custom-built thermostatic well plate with 45 individual wells containing 3 ml of 15‰ saltwater at 30°C. The well plate was filmed from above with a digital camera (Basler aCA1300-60gm, fitted with 5–50 mm, F1.4, CS mount lenses). Backlighting from an LED light board (Huion A4 LED light pad, set to maximum intensity) was used to provide contrast between the animals and the background. Video recording was stopped when a visual inspection indicated that all individuals were motionless. Individuals were then frozen overnight and weighed the next day. Mean±s.d. body mass was 54.8±22.9 mg, and did not differ between food treatments (fed: 56.1±23.5 mg, unfed: 53.2±22.1 mg, *P*=0.315). All experiments were carried out in agreement with EU legislation. To assess thermal tolerance, the resulting video files were processed in Ethovision (version XT 11.5, Noldus Information Technology, The Netherlands) to produce a time series of velocity data (in mm s^−1^, traveled by the center-point of each individual). Time to immobilization, *T*, was then estimated from the video-derived tracking data, using an algorithm in the R computing environment (https://www.r-project.org/) which objectively identifies the loss of locomotory function (Burton et al., 2020) from the tracking data (Dataset 1). Variation in *T* was analyzed using a linear model (LM) in R, with run, acclimation time, food treatment and centered body mass as predictor variables, including a three-way and all potential two-way interactions between the latter three variables. The full model was compared against all possible simpler models using the corrected Akaike information criterion (AICc).

### Estimating rate and capacity of plasticity

The rate of and capacity for plasticity in thermal tolerance was estimated following the method of Einum and Burton (2025). We first rescaled the time to immobilization data to have a mean of 0 at the first measurement timepoint for both food treatments, and with a subsequent increase, that is:
(1)

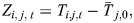
where *Z*_*i*, *j*, *t*_ is the rescaled time to immobilization for individual *i* in food treatment *j* at time *t*. We then fitted a non-linear model to the data:
(2)

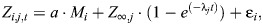
where *M* is the centered body mass, *Z*_∞_ is the rescaled asymptotic value of *T* when acclimation is complete (i.e. plasticity capacity), λ is the plasticity rate and ε is the residual variation. Thus, in this model we estimate a single effect of body mass on thermal tolerance, and different values for plasticity capacity and rate for the two food treatments. We then fitted alternative, less complex versions of this model, where we removed the effect of body mass and/or made plasticity capacity and/or rate independent of food treatment. The models were fitted using the *nls* function in R (v. 4.3.1), and compared using AICc. Figures were made using the *ggplot2* package (Wickham, 2016).

## RESULTS AND DISCUSSION

**Fig. 1.**
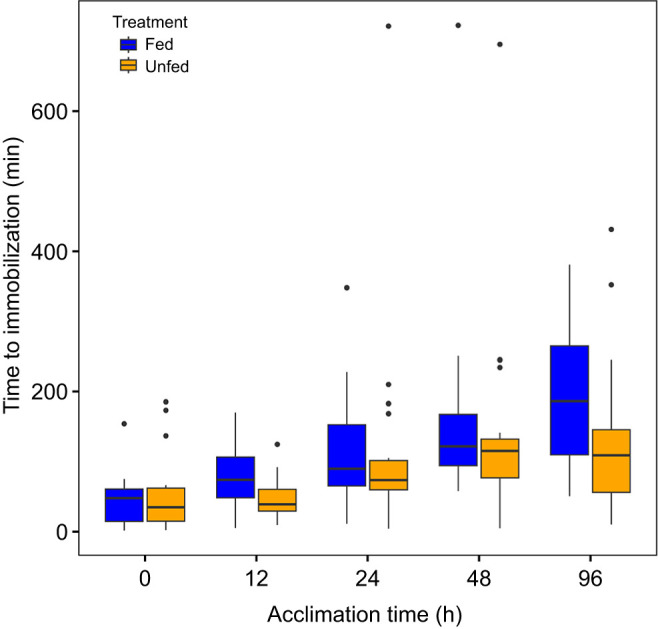
**Time to immobilization of *Echinogammarus marinus* when exposed to 30°C as a function of duration of acclimation to 20°C (shifted from 10°C) and feeding regime.** *N*=138 fed and 115 unfed individuals. Boxes represent the 25th and 75th percentiles, whiskers represent 1.5 interquartile range from the box, and points are observations outside this range.

The observed mean thermal tolerance increased with increasing duration of acclimation to warm temperature, and this increase was more pronounced for the fed than for the unfed treatment (Fig. 1; Table S1). According to the best model, the response to acclimation in the fed group was approximately twice as strong as that in the unfed group (Table S1).

**Fig. 2.**
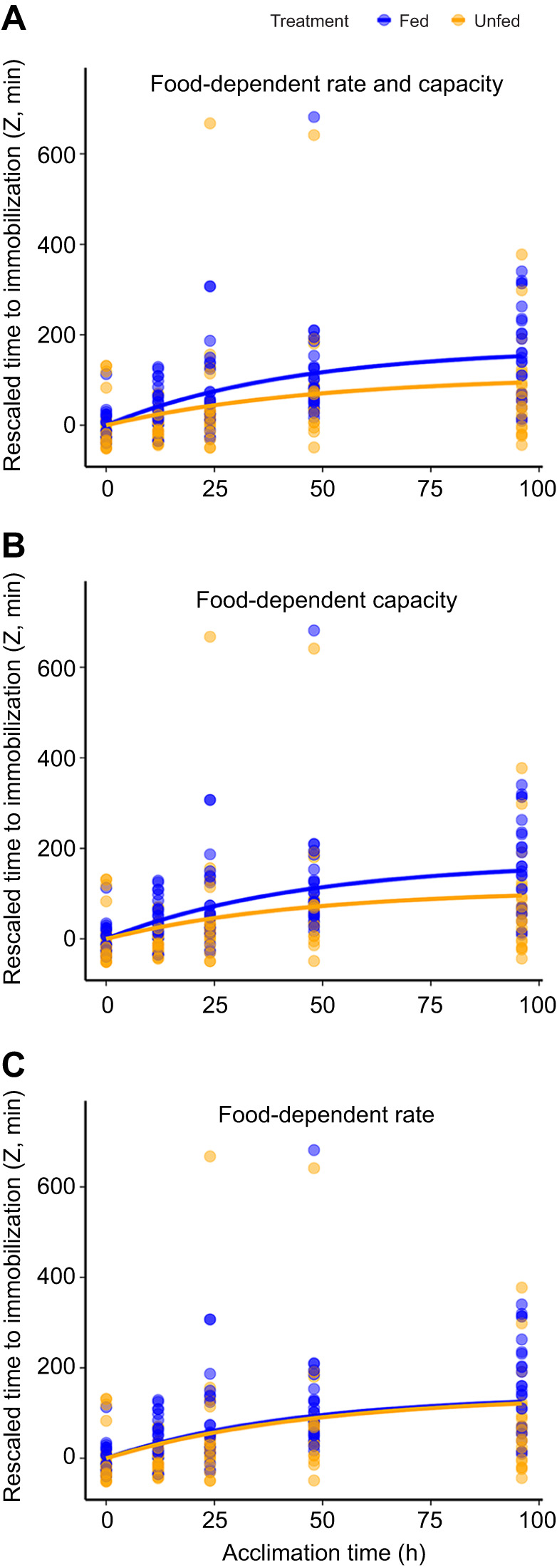
**Time to immobilization (rescaled, Eqn 1) in *E. marinus* when exposed to 30°C after different durations of acclimation to 20°C for individuals that were fed (*N*=138) or unfed (*N*=115) for the last 96 h prior to exposure.** Dots and error bars represent mean values±1 s.e. Fitted lines are predictions for individuals of mean body mass using the model-averaged parameter estimates for (A) food treatment dependent rate of and capacity for plasticity (as in Table 1B), (B) food treatment dependent capacity for plasticity but a common rate of plasticity (mean of food treatments: 0.022 h^−1^) and (C) food treatment dependent rate of plasticity but a common capacity for plasticity (140.4 min).

The analyses of rescaled (Eqn 1) time to immobilization identified an overall effect of food treatment on the temporal dynamics of the response in thermal tolerance to the change in temperature (Fig. 2A). Indeed, when comparing the different models, the top six models all contained an effect of treatment on plasticity capacity and/or rate, and the ΔAICc of the best model without such a term was 11.1 (model 7, Table 1). Three alternative models had a ΔAICc value of less or equal to 2. Two of these (models 1 and 3) included an effect of food treatment on the capacity for plasticity, whereas one (model 2) included an effect of food treatment on the rate of plasticity. All of the three alternative models that had a ΔAICc value of less or equal to 2 also included an effect of body mass on thermal tolerance. Owing to the lack of a single best model based on AICc values, we calculated parameter estimates and associated standard errors by averaging the complete set of models using Akaike weights. For the model averaging, parameter estimates for the body mass effect were set to 0 for models that lacked this term, whereas the estimates for plasticity rate and plasticity capacity for the two food treatments were set to the same estimated value for models that lacked the respective food treatment effects. Model-specific variances of the parameters were used to calculate the model-averaged variances and standard errors. The resulting model-averaged estimates showed a considerable difference in the capacity for plasticity between the two food treatments, with *T* being 57% lower in the unfed treatment relative to the fed treatment (Table 2). The change in the rate of plasticity in *T* was in the same direction, being lower in the unfed than in the fed treatment. However, this effect was considerably smaller in magnitude (10% difference, Table 2). Finally, there was a negative effect of body mass on thermal tolerance (Table 2).

**Table 1.** AICc and Akaike weights of alternative non-linear models (Eqn 2) explaining variation in thermal tolerance of *Echinogammarus marinus*

Model	Predictor terms	AICc	ΔAICc	*w_i_*
1	Body mass+capacity×treatment+rate	3013.3	0	0.51
2	Body mass+capacity+rate×treatment	3015.0	1.7	0.22
3	Body mass+capacity×treatment+rate×treatment	3015.3	2.0	0.19
4	Capacity×treatment+rate	3018.1	4.8	0.05
5	Capacity+rate×treatment	3019.9	6.6	0.02
6	Capacity×treatment+rate×treatment	3020.1	6.8	0.02
7	Body mass+capacity+rate	3024.4	11.1	<0.01
8	Capacity+rate	3026.9	13.6	<0.01

Interaction terms between capacity/rate and treatment indicate models where separate parameters are estimated for two different food treatments.

**Table 2.** Model-averaged parameter estimates describing variation in thermal tolerance for fed and unfed individuals of *E. marinus*

Parameters	Estimate	s.e.
Capacity, fed	171.6	35.0
Capacity, unfed	109.1	27.6
Rate, fed	0.023	0.010
Rate, unfed	0.021	0.012
Body mass (mg)	−0.62	0.25

To examine the relative role of food treatment effects on rate versus capacity in shaping the temporal dynamics of the plastic response, we used the model-averaged estimates to generate two sets of predicted data. First, we used the estimates of the capacity for plasticity for each food treatment, together with a common estimate for plasticity rate that was independent of food treatment (i.e. the mean of the two rate estimates; Table 2). The temporal dynamics of thermal tolerance for these predictions (Fig. 2A) was virtually indistinguishable from the predictions from the model where both rate and capacity depended on food treatment (Fig. 2B). Second, we used the food treatment dependent estimates for plasticity rate but a common estimate for plasticity capacity (i.e. the mean of the two capacity estimates; Table 2). For these predictions, the effect of food treatment on plasticity became negligible (Fig. 2C). Thus, the effect of food treatment on the temporal dynamics of thermal tolerance was primarily driven by effects on the capacity for plasticity.

Phenotypic plasticity enables organisms to withstand fluctuating environments and associated changes in trait optima. However, environments are multidimensional, and phenotypic plasticity may therefore be best understood by simultaneous consideration of two or more of these dimensions (Westneat et al., 2019). We tested for the effects of food availability and thermal acclimation on thermal tolerance in the amphipod *E. marinus*. Our results show that this species upregulates thermal tolerance when acclimated to a sub-lethal warm temperature, but that this response is compromised when energy intake is limited. This suggests that the plastic response to temperature change entails an energy cost that is not completely paid in the absence of food. Further, we provide the first attempt to examine the effects of food availability on the rate and capacity components of phenotypic plasticity. We observed that the weaker plastic response to temperature change in the absence of food was caused primarily by a reduced capacity for plasticity. In comparison, the effect of food deprivation on the rate of plasticity contributed little to the overall dynamics of the plasticity response to temperature change.

Our study sheds light on the issue of costs of plasticity. Such costs can broadly be categorized into maintenance and production costs (Auld et al., 2010). Maintenance costs are those that are paid to build and maintain the ability to detect and respond phenotypically to changes in the environment. Such costs are paid continuously throughout life of the organism, independently of the environmental conditions that they experience. Therefore, it is this type of cost that is presumed to select against plasticity under stable environmental conditions (Sultan and Spencer, 2002). In contrast, production costs are those that are paid to change the phenotype, and they are thus only incurred once an environmental change is detected and are presumably offset by the benefits yielded by plasticity. In the present study, it seems unlikely that acute food deprivation over 96 h caused developmental effects on the machinery responsible for detecting and responding to changes in the environment. Thus, a more immediate effect on the ability to cover production costs may be more likely to have resulted from this treatment, and this appeared to primarily impact the capacity for plasticity. The lack of an effect of such short-term food deprivation on plasticity rate supports the hypothesis that the rate of plasticity may be more sensitive to the size of the underlying machinery required for phenotypic change rather than the immediate availability of energy (Burton et al., 2022). If maintenance costs are more relevant for the rate component of plasticity, and production costs more important for the capacity component, one might hypothesize that a more chronic food deprivation throughout development could have implications for the rate of plasticity; however, this remains to be tested. Furthermore, gammarids are known to accumulate substantial levels of lipids (Baeza-Rojano et al., 2014) that may be utilized to compensate for short-term food deprivation, and thus the results obtained here may differ for organisms that lack this option.

Our study emphasizes the potential importance of ecological context for how efficiently organisms may cope with temperature fluctuations. Specifically, energy limitation may considerably constrain the ability of organisms to acclimate to high temperatures owing to reduced plasticity capacity, and this may reduce their probability of survival when exposed to extreme temperature. Energy limitation may arise via different mechanisms, with their relative importance depending on the time scale considered. In the short term, sustained exposure to sub-lethal warm temperature (but above the optimum) may depress individuals' food consumption even if food is freely available (Sinclair et al., 2016). An impaired ability to manifest plasticity may then be particularly damaging because in a stochastic autocorrelated environment it is during such periods that the occurrence of more extreme lethal temperatures is more likely. In the longer term, changes in thermal regimes may be accompanied by changes in community composition (prey, competitors, parasites; Feeley et al., 2020; Cremona et al., 2020), which may in turn impact the energetic status of individuals. Such longer-term ecological effects may be relevant when considering how climate change is increasing both the mean and variance of temperature (Van der Wiel and Bintanja, 2021), where increased mean temperature could drive community change, and at the same time increased variance will increase the probability of lethal extreme temperatures. Finally, if empirical temperature thresholds of species are applied in conservation management decisions or for predicting distributional changes (e.g. Huey et al., 2012; Marchessaux et al., 2024), care should be taken to consider not only how phenotypic plasticity might shift thermal tolerance when setting such thresholds (Somero, 2010; Huey et al., 2012; Verberk et al., 2023), but also how ecological conditions might impair the ability of organisms to produce plastic changes in the phenotype. Laboratory derived thermal tolerance data from experiments where ecological conditions are otherwise optimal may provide overly optimistic estimates of how well organisms deal with extreme events through phenotypic plasticity.

## Supplementary Material

10.1242/jexbio.251713_sup1 Supplementary information

Dataset 1.
