# Supplementary material for: Energy availability influences the dynamics of thermal phenotypic plasticity
Source: J Exp Biol. 2026 Jan 14;229(2):jeb251713. doi: 10.1242/jeb.251713 (PMC12863295; doi:10.1242/jeb.251713)
Supplement: Supplementary information [file jexbio-229-251713-s1.pdf]

Table. S1. (A) AICc and Akaike weights of alternative linear models explaining variation in thermal tolerance of *E. marinus* as a function of experimental run (R), body mass (BM), food treatment (FT) and acclimation time (AT). Only models with ΔAICc < 5 are shown. All three models with ΔAICc < 2 contained an interaction term between acclimation time and food treatment, giving strong support for such an interaction. Inspection of residuals from the best model showed no evidence for any strongly influential data points, with the maximum Cook's distance being 0.13. (B) Parameter estimates for the top model describing variation in thermal tolerance for fed and unfed individuals of *E. marinus*.

**(A) Predictor terms**	**AICc**	**ΔAICc**	w_i
BM + FT × AT	3021.7	0.00	0.196
FT × AT + BM × AT	3022.4	0.63	0.143
BM + FT × AT + R	3022.9	1.16	0.110
BM × FT + AT × FT	3023.9	2.11	0.068
BM × AT + AT × FT + R	3023.9	2.15	0.067
BM + FT + AT	3024.3	2.56	0.055
BM × FT + BM × AT + AT × FT	3024.5	2.76	0.049
BM × FT + AT × FT + R	3025.1	3.37	0.036
BM × AT + FT	3025.3	3.55	0.033
BM + FT + AT + R	3025.5	3.71	0.031
BM × FT × AT	3025.6	3.85	0.029
BM + AT	3026.1	4.32	0.023
BM × FT + BM × AT + AT × FT + R	3026.1	4.35	0.022
BM × FT + AT	3026.4	4.60	0.020
BM + R + AT	3026.5	4.77	0.018

**(B) Parameters**	**Estimate**	**SE**

Journal of Experimental Biology • Supplementary information

Intercept	62.60	11.73
Body mass (mg)	-0.72	0.26
Treatment (unfed)	3.66	17.25
Acclimation time (h)	1.54	0.24
Acclimation time × food treatment (unfed)	-0.77	0.36

Script 1. R code for analyses of data

```
library(ggplot2)
library(propagate)
library(boot)
library(minpack.lm)
library(cowplot)
library(AICcmodavg)
library(MuMIn)
library(dplyr)

data <- read.delim("path_to_data/thermal_tolerance_data.csv", sep=";")

data$Treatment <- as.factor(data$Treatment)
data$Run <- as.factor(data$Run)
mean(data$Weight)
sd(data$Weight)
hist(data$Weight)
min(data$Weight)
max(data$Weight)

#test for difference in body mass between treatments
tapply(data$Weight, data$Treatment, mean)
```

Journal of Experimental Biology • Supplementary information

```
tapply(data$Weight, data$Treatment, sd)
mod <- lm(data$Weight~data$Treatment)
summary(mod)

#figure raw data
Fig1 <- ggplot(data = data, aes(x = as.factor(Acclim_time), y = (Timm), fill = Treatment)) +
 geom_boxplot() +
 xlab("\nAcclimation time (h)") +
 ylab("Time to immobilization (min)") +
 scale_fill_manual(values = c("Fed" = "blue", "Unfed" = "orange")) +  # Adjust color if needed
 theme_bw(base_size = 20) +
 theme(panel.grid.major = element_blank(), panel.grid.minor = element_blank()) +
 theme(panel.border = element_rect(colour = "black")) +
 theme(axis.ticks = element_line(color = "black")) +
 theme(axis.text = element_text(color = "black")) +
 theme(axis.text.y = element_text(hjust = 0.5))

ggsave("", width = 250, height = 200, units = "mm", Fig1, dpi = 600)

#centre body mass in mg
data$Weight <- 1000*(data$Weight - mean(data$Weight))

#analyses of variance in timm
str(data)
mod <- lm(Timm ~ Run + Acclim_time*Treatment*Weight, data=data, na.action=na.fail)
dredge(mod)

mod <- lm(Timm ~ Weight+Acclim_time*Treatment, data=data, na.action=na.fail)
summary(mod)
max(cooks.distance(mod))
```

Journal of Experimental Biology • Supplementary information

```
#Rescaling data

data$Zt <- NA

data$Zt <- ifelse(data$Treatment =="Fed", data$Timm - mean(data$Timm[data$Acclim_time ==
0 & data$Treatment == "Fed"]),data$Timm - mean(data$Timm[data$Acclim_time == 0 &
data$Treatment == "Unfed"]) )


data$Run <- as.factor(data$Run)


#fit different models with treatment effects on capacity and/or rate

m1 <- nls(Zt ~ a*Weight + Zinf*(1-exp(-lambda * Acclim_time)),
     data = data,
     start = list(Zinf = 100, lambda = 0.1, a = -1), na.action=na.fail)


m2 <- nls(Zt ~ a*Weight+ Zinf[Treatment]*(1-exp(-lambda * Acclim_time)),
     data = data,
     start = list(Zinf = rep(100,2), lambda = 0.01, a = -1))


m3 <- nls(Zt ~ a*Weight+ Zinf*(1-exp(-lambda[Treatment] * Acclim_time)),
     data = data,
     start = list(Zinf = 100, lambda = rep(0.01,2), a = -1))


m4 <- nls(Zt ~ a*Weight+ Zinf[Treatment]*(1-exp(-lambda[Treatment] * Acclim_time)),
     data = data,
     start = list(Zinf = rep(100,2), lambda = rep(0.01,2), a = -1))


m5 <- nls(Zt ~ Zinf*(1-exp(-lambda * Acclim_time)),
     data = data,
     start = list(Zinf = 100, lambda = 0.1), na.action=na.fail)


m6 <- nls(Zt ~ Zinf[Treatment]*(1-exp(-lambda * Acclim_time)),
     data = data,
```

Journal of Experimental Biology • Supplementary information

```
     start = list(Zinf = rep(100,2), lambda = 0.01))

m7 <- nls(Zt ~ Zinf*(1-exp(-lambda[Treatment] * Acclim_time)),
     data = data,
     start = list(Zinf = 100, lambda = rep(0.01,2)))

m8 <- nls(Zt ~ Zinf[Treatment]*(1-exp(-lambda[Treatment] * Acclim_time)),
     data = data,
     start = list(Zinf = rep(100,2), lambda = rep(0.01,2)))



models <- list(m1,m2,m3,m4,m5,m6,m7,m8)
aicc_values <- sapply(models, AICc)

delta_aicc <- aicc_values - min(aicc_values)
weights <- exp(-0.5 * delta_aicc) / sum(exp(-0.5 * delta_aicc))

# Create a function to pad and duplicate coefficients
pad_and_duplicate_coefficients <- function(coefs, all_names) {
  padded <- rep(0, length(all_names))  # Initialize with 0
  names(padded) <- all_names
  for (name in names(coefs)) {
    if (name %in% all_names) {
      padded[name] <- coefs[name]
    } else {
      # Handle single estimates by duplicating them
      if (name == "lambda") {
        padded["lambda1"] <- coefs[name]
        padded["lambda2"] <- coefs[name]
      } else if (name == "Zinf") {
```

Journal of Experimental Biology • Supplementary information

```
        padded["Zinf1"] <- coefs[name]
        padded["Zinf2"] <- coefs[name]
      }
    }
  }
  return(padded)
}

# Get all unique parameter names
all_names <- c("Zinf1", "Zinf2", "lambda1", "lambda2", "a")

# Pad and duplicate coefficients for each model
coefficients <- lapply(models, coef)
padded_coefficients <- lapply(coefficients, pad_and_duplicate_coefficients, all_names =
all_names)

# Calculate model-averaged coefficients
model_avg_coefficients <- sapply(seq_along(all_names), function(i) {
  coefs <- sapply(padded_coefficients, `[`, i)
  sum(coefs * weights, na.rm = TRUE)
})
names(model_avg_coefficients) <- all_names

# Calculate the variance-covariance matrices for each model
vcov_matrices <- lapply(models, vcov)

# Create a function to pad and duplicate variance-covariance matrices
pad_and_duplicate_vcov <- function(vcov_matrix, all_names) {
  padded <- matrix(0, nrow = length(all_names), ncol = length(all_names))
  rownames(padded) <- all_names
  colnames(padded) <- all_names
```

Journal of Experimental Biology • Supplementary information

```
 for (name1 in rownames(vcov_matrix)) {
  for (name2 in colnames(vcov_matrix)) {
   if (name1 %in% all_names && name2 %in% all_names) {
    padded[name1, name2] <- vcov_matrix[name1, name2]
   } else {
    if (name1 == "lambda" && name2 == "lambda") {
     padded["lambda1", "lambda1"] <- vcov_matrix[name1, name2]
     padded["lambda2", "lambda2"] <- vcov_matrix[name1, name2]
    } else if (name1 == "Zinf" && name2 == "Zinf") {
     padded["Zinf1", "Zinf1"] <- vcov_matrix[name1, name2]
     padded["Zinf2", "Zinf2"] <- vcov_matrix[name1, name2]
    }
   }
  }
 }
 return(padded)
}

# Pad and duplicate variance-covariance matrices for each model
padded_vcov_matrices <- lapply(vcov_matrices, pad_and_duplicate_vcov, all_names =
all_names)

# Calculate the model-averaged variance-covariance matrix
model_avg_vcov <- Reduce("+", Map("*", padded_vcov_matrices, weights))

# Extract the standard errors from the diagonal of the model-averaged variance-covariance
matrix
model_avg_se <- sqrt(diag(model_avg_vcov))
names(model_avg_se) <- all_names

# Print model-averaged coefficients and their standard errors
print(model_avg_coefficients)
```

Journal of Experimental Biology • Supplementary information

```
print(model_avg_se)


##################################################FIGURES WITH PREDICTIONS

#Figure with predictions from modelaverage
#make predictions
# Define a sequence of Acclim_time values for predictions
pred_time <- seq(min(data$Acclim_time), max(data$Acclim_time), length.out = 100)
data_fed <- data[data$Treatment == "Fed",]
data_unfed <- data[data$Treatment == "Unfed",]
#generate predictions
pred_m2_fed <- model_avg_coefficients[5]*mean(data$Weight)+ model_avg_coefficients[1]*(1-exp(-model_avg_coefficients[3] * pred_time))
pred_m2_unfed <- model_avg_coefficients[5]*mean(data$Weight)+ model_avg_coefficients[2]*(1-exp(-model_avg_coefficients[4] * pred_time))

# Combine predictions into a data frame
pred_data <- data.frame(
  Acclim_time = rep(pred_time, 2),
  Zt = c(pred_m2_fed, pred_m2_unfed),
  Treatment = rep(c("Fed", "Unfed"), each = length(pred_time))
)


# Plot the data points and predicted lines
Fig2a <- ggplot(data, aes(x = Acclim_time, y = Zt, color = Treatment)) +
  geom_point(size = 5, alpha = 0.5) +
  geom_line(data = pred_data, aes(x = Acclim_time, y = Zt, color = Treatment), linewidth = 2) +
  annotate("text", x = 50, y = 750, label = "Food dependent rate and capacity", size = 10) +  # Add text annotation
  labs(x = "", y = "Rescaled time to immobilization (Z, min)") +
```

Journal of Experimental Biology • Supplementary information

```
 theme_minimal(base_size= 25) +
 scale_color_manual(values = c("Fed" = "blue", "Unfed" = "orange")) +
 scale_fill_manual(values = c("Fed" = "blue", "Unfed" = "orange")) +
 theme(axis.ticks = element_line(linewidth = 0.5),  # Adjust tick size
    axis.ticks.length = unit(0.2, "cm"))+   # Adjust tick length
 theme(
  panel.grid = element_blank(),  # Remove background grid
  axis.line = element_line(color = "black"),  # Add x- and y-axis lines
  legend.position = "top",  # Move legend to the top
  legend.justification = c(1, 1)  # Adjust legend to the top right corner
 )

##############################Figure with predictions when assuming no effect on rate, only effect on capacity
#make predictions
# Define a sequence of Acclim_time values for predictions
pred_time <- seq(min(data$Acclim_time), max(data$Acclim_time), length.out = 100)
data_fed <- data[data$Treatment == "Fed",]
data_unfed <- data[data$Treatment == "Unfed",]

mean_rate <- (model_avg_coefficients[3]+model_avg_coefficients[4])/2

#generate predictions
pred_m2_fed <- model_avg_coefficients[5]*mean(data$Weight)+ model_avg_coefficients[1]*(1-exp(-mean_rate * pred_time))
pred_m2_unfed <- model_avg_coefficients[5]*mean(data$Weight)+ model_avg_coefficients[2]*(1-exp(-mean_rate * pred_time))

# Combine predictions into a data frame
pred_data <- data.frame(
 Acclim_time = rep(pred_time, 2),
 Zt = c(pred_m2_fed, pred_m2_unfed),
```

```
  Treatment = rep(c("Fed", "Unfed"), each = length(pred_time))
)


# Plot the data points and predicted lines
Fig2b <- ggplot(data, aes(x = Acclim_time, y = Zt, color = Treatment)) +
  geom_point(size = 5, alpha = 0.5) +
  geom_line(data = pred_data, aes(x = Acclim_time, y = Zt, color = Treatment), linewidth = 2) +
  annotate("text", x = 50, y = 750, label = "Food dependent capacity", size = 10) +  # Add text annotation
  labs(x = "", y = "Rescaled time to immobilization (Z, min)") +
  theme_minimal(base_size= 25) +
  scale_color_manual(values = c("Fed" = "blue", "Unfed" = "orange")) +
  scale_fill_manual(values = c("Fed" = "blue", "Unfed" = "orange")) +
  theme(axis.ticks = element_line(linewidth = 0.5),  # Adjust tick size
        axis.ticks.length = unit(0.2, "cm"))+   # Adjust tick length
  theme(
    panel.grid = element_blank(),  # Remove background grid
    axis.line = element_line(color = "black"),  # Add x- and y-axis lines
    legend.position = "none",  # Move legend to the top
    legend.justification = c(1, 1)  # Adjust legend to the top right corner
  )


##############################Figure with predictions when assuming no effect on capacity, only effect on rate
#make predictions
# Define a sequence of Acclim_time values for predictions
pred_time <- seq(min(data$Acclim_time), max(data$Acclim_time), length.out = 100)
data_fed <- data[data$Treatment == "Fed",]
data_unfed <- data[data$Treatment == "Unfed",]
```

Journal of Experimental Biology • Supplementary information

```
mean_capacity <- (model_avg_coefficients[1]+model_avg_coefficients[2])/2

#generate predictions
pred_m2_fed <- model_avg_coefficients[5]*mean(data$Weight)+ mean_capacity*(1-exp(-
model_avg_coefficients[3] * pred_time))
pred_m2_unfed <- model_avg_coefficients[5]*mean(data$Weight)+ mean_capacity*(1-exp(-
model_avg_coefficients[4] * pred_time))

# Combine predictions into a data frame
pred_data <- data.frame(
 Acclim_time = rep(pred_time, 2),
 Zt = c(pred_m2_fed, pred_m2_unfed),
 Treatment = rep(c("Fed", "Unfed"), each = length(pred_time))
)


# Plot the data points and predicted lines
Fig2c <- ggplot(data, aes(x = Acclim_time, y = Zt, color = Treatment)) +
 geom_point(size = 5, alpha = 0.5) +
 geom_line(data = pred_data, aes(x = Acclim_time, y = Zt, color = Treatment), linewidth = 2) +
 annotate("text", x = 50, y = 750, label = "Food dependent rate", size = 10) +  # Add text
annotation
 labs(x = "Acclimation time (h)", y = "Rescaled time to immobilization (Z, min)") +
 theme_minimal(base_size= 25) +
 scale_color_manual(values = c("Fed" = "blue", "Unfed" = "orange")) +
 scale_fill_manual(values = c("Fed" = "blue", "Unfed" = "orange")) +
 theme(axis.ticks = element_line(linewidth = 0.5),  # Adjust tick size
    axis.ticks.length = unit(0.2, "cm"))+   # Adjust tick length
 theme(
  panel.grid = element_blank(),  # Remove background grid
  axis.line = element_line(color = "black"),  # Add x- and y-axis lines
```

Journal of Experimental Biology • Supplementary information

```
  legend.position = "none",  # Move legend to the top
  legend.justification = c(1, 1)  # Adjust legend to the top right corner
 )


# arrange the plots in a single row
Fig2 <- plot_grid(
 Fig2a,
 Fig2b,
 Fig2c,
 align = 'hv',
 axis = 'l',
 labels = c("A", "B", "C"),
 label_x = 0.15,
 label_y = 0.9,
 label_size = 30,
 hjust = -1,
 nrow = 3
)
Fig2
ggsave("", width = 200, height = 600, units = "mm", Fig2, dpi = 600)
```

Dataset 1.

Available for download at
https://journals.biologists.com/jeb/article-lookup/doi/10.1242/jeb.251713#supplementary-data

Journal of Experimental Biology • Supplementary information
